# Emotional barriers and facilitators of deprescribing for older adults with cancer and polypharmacy: a qualitative study

**DOI:** 10.1007/s00520-023-08084-9

**Published:** 2023-10-17

**Authors:** Erika Ramsdale, Arul Malhotra, Holly M. Holmes, Lisa Zubkoff, Jinjiao Wang, Supriya Mohile, Sally A. Norton, Paul R. Duberstein

**Affiliations:** 1 James P. Wilmot Cancer Center, University of Rochester Medical Center, 601 Elmwood Avenue, Box 704, Rochester, NY 14642 USA; 2 Division of Geriatric and Palliative Medicine, McGovern Medical School, Houston, TX USA; 3 Department of Medicine, University of Alabama at Birmingham, Birmingham, AL USA; 4 School of Nursing, University of Rochester Medical Center, Rochester, NY USA; 5 Department of Health Behavior, Society, and Policy, Rutgers School of Public Health, Piscataway, NJ USA

**Keywords:** Polypharmacy, Deprescribing, Focus groups, Pharmacists

## Abstract

**Purpose:**

To describe emotional barriers and facilitators to deprescribing (the planned reduction or discontinuation of medications) in older adults with cancer and polypharmacy.

**Methods:**

Virtual focus groups were conducted over Zoom with 5 key informant groups: oncologists, oncology nurses, primary care physicians, pharmacists, and patients. All groups were video- and audio-recorded and transcribed verbatim. Focus group transcripts were analyzed using inductive content analysis, and open coding was performed by two coders. A codebook was generated based on the initial round of open coding and updated throughout the analytic process. Codes and themes were discussed for each transcript until consensus was reached. Emotion coding (identifying text segments expressing emotion, naming the emotion, and assigning a label of positive or negative) was performed by both coders to validate the open coding findings.

**Results:**

All groups agreed that polypharmacy is a significant problem. For clinicians, emotional barriers to deprescribing include fear of moral judgment from patients and colleagues, frustration toward patients, and feelings of incompetence. Oncologists and patients expressed ambivalence about deprescribing due to role expectations that physicians “heal with med[ication]s.” Emotional facilitators of deprescribing included the involvement of pharmacists, who were perceived to be neutral, discerning experts. Pharmacists described emotionally aware communication strategies when discussing deprescribing with other clinicians and expressed increased awareness of patient context.

**Conclusion:**

Deprescribing can elicit strong and predominantly negative emotions among clinicians and patients which could inhibit deprescribing interventions. The involvement of pharmacists in deprescribing interventions could mitigate these emotional barriers.

**Trial registration:**

ClinicalTrials.gov Identifier: NCT05046171. Date of registration: September 16, 2021.

**Supplementary Information:**

The online version contains supplementary material available at 10.1007/s00520-023-08084-9.

## Introduction

Polypharmacy is the concurrent use of multiple medications and is highly prevalent in older adults with cancer. A recent analysis of older adults treated in community oncology settings showed 61.3% were on ≥5 regular medications and 14.5% were on ≥10 regular medications, in addition to their cancer treatment-related and supportive care medications [1]. Polypharmacy is more common in patients with cancer and cancer survivors than in community-dwelling patients without a cancer history [2, 3]. Polypharmacy is associated with adverse outcomes in older adults with cancer [4], including hospitalizations, decreased overall survival, chemotherapy toxicity, falls, and functional decline.

“Deprescribing” is the planned reduction or discontinuation of medications that may pose risk higher than benefit, supervised by a healthcare professional [5]. It has been proposed as a key intervention to reduce potential harms from polypharmacy. However, meta-analyses of deprescribing intervention trials have shown limited effectiveness in reducing adverse outcomes in older adults [6, 7]. Analyses are hampered by wide variability in deprescribing interventions, which range from simple educational interventions targeting prescribers to complex multi-disciplinary team interventions. One recent meta-analysis indicated that comprehensive medication review, typically performed by a pharmacist, may have the most effectiveness (compared to a range of other deprescribing interventions) in reducing adverse outcomes such as potentially inappropriate medication use and mortality [6]. Another meta-analysis suggested a reduction in adverse outcomes for personalized (patient-specific) deprescribing approaches, versus no reduction for general education interventions like educating clinicians on deprescribing [8]. However, there are few studies investigating deprescribing in older adults with cancer [9], and studies that have enrolled these patients have been largely focused on end-of-life deprescribing [10, 11].

Deprescribing can be considered an example of “de-implementation,” which poses broad challenges for patients, clinicians, and organizations. De-implementation involves the discontinuation of practices that are ineffective, less effective than alternative practices, or potentially harmful [12]. Compared to implementation of a tested intervention, de-implementation induces doubt, confusion, fear, and aversion, and therefore involves unique emotional and cognitive barriers [13, 14]. Although most patients are willing to stop medications if their physician recommends it [15, 16], deprescribing requires more time and effort than that involved in prescribing the medication. Two thirds of outpatient visits in the USA involve prescribing a medication [17], and one study reported the average time spent discussing a new medication was 49 s (5% of total visit time) [18]. In contrast, assessing the risks and benefits of each medication in the context of the patient’s current situation, a precursor to deprescribing, can require 10–15 min or more [9]. In modern allopathic medicine, prescribing a pill to address a concern is an expected interaction in the clinic by both patients [19, 20] and care providers [21, 22], exposing a strong cultural and scientific preference for addressing tractable problems with straightforward solutions (i.e., a pill) [23] over difficult, intractable problems inherent to aging, illness, and disability [24]. Systems barriers such as fragmented care [22] and poor electronic health record interoperability [25] further hinder deprescribing. Although significant attention is paid to addressing care fragmentation and interoperability, structural changes alone may not address a medical culture that has created moral distress in providers and care teams by creating time pressure, emphasizing “productivity,” and prioritizing number of patients seen and procedures completed over quality and comprehensiveness of care [26].

As part of a clinical trial investigating deprescribing interventions for older adults starting chemotherapy (Decreasing Polypharmacy in Older Adults With Curable Cancers Trial, PI: Ramsdale, ClinicalTrials.gov Identifier: NCT05046171) focus groups were conducted to elicit barriers and facilitators to deprescribing. Although barriers and facilitators to deprescribing have been described previously [27–29], we are aware of no prior research on emotional barriers to deprescribing in older adults with cancer. Insufficient attention to emotional and behavioral nuances rooted in a particular culture of medicine or cancer care could limit dissemination and sustainability of deprescribing, even if other barriers are addressed. In this paper, emotional resistance to deprescribing is explored.

## Methods

### Study design and participants

Virtual focus groups were conducted over Zoom with 5 key informant groups: oncologists (*n*=6), oncology nurses (*n*=7), primary care physicians (*n*=7), pharmacists (*n*=7), and patients (*n*=9). All focus groups occurred between November 2020 and August 2021. These participant groups were selected based on involvement in evaluating or experiencing medication usage for older adults seen in the oncology clinic. Oncologists were recruited via email to eligible individuals from University of Rochester Wilmot Cancer Institute, its community affiliate Interlakes Oncology, and the National Cancer Institute (NCI) Community Oncology Research Program (NCORP) community affiliate sites. Oncologist participants were eligible if they were in active clinical practice at one or more of the above locations. Oncology nurses, primary care physicians, and pharmacists were recruited via email to eligible individuals from the University of Rochester Medical Center. All individuals in active employment as an oncology nurse, primary care physician, or pharmacist were eligible. Patients were recruited from the Stakeholders for Care in Oncology & Research for our Elders Board (SCOREBoard) [30], which is the patient advisory board for the Cancer and Aging Research Group (CARG). SCOREBoard participants did not have formal training about deprescribing. The protocol was reviewed and approved by the University of Rochester Institutional Review Board, and all participants provided informed consent. Participants were not paid for participation in the focus group activities.

Focus groups were conducted by trained moderators (EC or JB, both research coordinators) with a second moderator (ER, the principal investigator and a practicing geriatric oncologist) observing all groups and taking contemporaneous notes. All groups were video- and audio-recorded and transcribed verbatim. Focus group discussion lasted an average of 54 min (range 44–62 min). A semi-structured interview guide was tailored to each group using “funnel” (moving from broad topics to narrower ones) and “reverse funnel” (starting with a narrow topic and broadening the focus) approaches to facilitate coverage of all relevant topics related to polypharmacy in older adults and deprescribing (Supplemental Appendix) [31]. Interview guide topics included definitions of polypharmacy, current medication-related workflows, communication about medications, and approaches to deprescribing. An introduction to the focus group introduced the reason for the research, to gather perspectives on polypharmacy and deprescring as part of a NCI-funded trial. The PI (ER) is a colleague of most of the participants. The focus group participants had not previously met or interacted with the other moderators.

### Analysis

Debriefing sessions were held with moderators following each group for comparing notes and impressions. Focus group transcripts were analyzed using inductive content analysis [32]. Open coding was performed by the principal investigator (ER) and one additional coder (AM). A codebook was generated based on the initial round of open coding and updated throughout the analytic process. Codes and themes were discussed for each transcript until consensus was reached. A summary of key themes and interpretations for each group was sent to the group participants for comment via email (member checking) [33]. Based on a notable and surprising theme of emotional resistance to deprescribing identified in the open coding, we made a decision to recode the data using emotion coding [34] to explore this unexpected phenomenon in more depth. Emotion coding consisted of identifying text segments expressing emotion, naming the emotion (e.g., frustration, anger, gratitude) and assigning a label of positive or negative. Portions of the transcript not expressing positive or negative emotion were considered neutral. Emotion coding was performed by ER and one additional coder, with discrepancies reviewed and discussed until consensus was reached. All analyses were completed using MAXQDA 2020 software. The COnsolidated criteria for REporting Qualitative research (COREQ) checklist was used and is included in the supplemental appendix [35].

## Results

Analysis revealed agreement by clinicians and patients that polypharmacy was a problem requiring investigation and intervention. There were no statements in any of the focus groups indicating low prioritization or lack of recognition of polypharmacy.

Patient 1: “But many times, people are taking medications that they've just been taking for years and they don't need them anymore, or they've become harmful.” Patient 2: “I agree.” (Patients)

“I think it’s a real problem.…[s]o yeah, polypharmacy is on the problem list. And I think it’s a diagnosis. And really much more dangerous in an elderly population than somebody young.” (Community oncologist)

Despite these statements, discussion about how to address polypharmacy and implement deprescribing elicited multiple barriers and facilitators; this paper focuses on those eliciting emotion as evidenced by use of semantics, tone, or use of sarcasm.

### Emotional barriers to deprescribing

Although participants recognized polypharmacy as a problem, prompts to discuss how to explicitly implement deprescribing elicited emotional barriers to deprescribing, including expressions of fear, shame, anxiety, doubt, confusion, and frustration (Table 1 and Supplemental Table 1). Two subthemes related to emotional barriers overlapped within the oncologist, primary care physician (PCP), and pharmacist groups:

1. Clinicians were *apprehensive* about reactions and moral judgments from colleagues, including being judged as “stupid” or disrespectful of other clinicians’ expertise.
2. Clinicians felt *preoccupied* about the reactions and moral judgments from patients, such as patients feeling abandoned or angry.

**Table 1:** Emotional barriers to deprescribing

*Subtheme*	*Group*					*Example quotes*
	Onc	PCP	Nurse	Pharm	Pts	
Apprehensive about colleague reactions	X	X		X		• “When I’m interacting with a pulmonologist or cardiologist, I’m like, I say do you really think we need that propranolol and they’re like well – you don't know how they are going react. You know, what do you know, you’re just the internist. Or of course they need that! And then you feel stupid…”(*PCPs*) • “There’s probably a limit to how interested the primary care doctor is to hearing my opinion on their lisinopril dose. Okay, buddy, I just want to know why the white count is 12, lay off.” (*Oncologist*)
Preoccupied with patient reactions	X	X		X		• “I also think that there is suspicion for abandonment with patients. They might think you have a different agenda other than trying to help them so I try to make that clear, that a number of years ago when I took the Hippocratic oath to do no harm I took that seriously.” (*PCP)* • “Sometimes the providers don't feel comfortable or know exactly how to approach that conversation with the patient, just because for so long we were wanting to get these patients on those medications and so now to have kind of the opposite conversation where we're like, ‘Oh you don't need this anymore,’ it's difficult to have the finesse for that conversation without it sounding like we're just giving up on you because you're old.” (*Pharmacist)*
Frustration toward patients	X	X	X			• “I think all of us but especially geriatric patients are very emotionally tied to their medications…some people are more committed to their omeprazole than to their wife.” (*Nurse*) • “No matter how many times we ask the patients to just bring all the bottles in, I’ll write them down, you know, bring in everything you’ve taken in the last week, just bring it to me, they don’t necessarily do it. They don’t understand.” (*Oncologist*)
Uncertain about competence	X	X	X			• “I wasn’t in the room when the oncologist was explaining everything. You know? They’re the one with the white coat. Not me. I’m a nurse in scrubs.” (*Nurse*) • “There are people that come in and I don't feel competent…first of all to understand why they are on some of these medications and whether I can stop them or not.” (*PCP*)
Overwhelmed and confused by roles in a fragmented system	X	X	X			• “So who is making it? Is this oncology’s responsibility? The provider? Who is the one taking control over all these medications? Because generally all of these medications wouldn’t be prescribed by the oncology team necessarily. Ah, actually…I don't know. But yeah, a lot of questions and a lot of emotion.” (*Nurse*) • “All of us get so many communications in a day that it does become overwhelming of how to filter out what’s important and what’s not and what do I need to address and what’s not my responsibility, what’s somebody else’s responsibility and that can be a challenge.” (*Oncologist*)
Ambivalence about deprescribing based on expected role	X				X	• “So that’s kind of the way we are taught, is that you heal with meds.” (*Oncologist*) • *Patient 1:* “And I find that often doctors suggest taking a medication because it might be the newest, but they don't know much about it…” *Patient 2*: Or they just give it to you because it's protocol.” (*Patients*)

Three further subthemes were identified from the oncologist, PCP, and nurse groups, but not the pharmacist group:

1. Clinicians exhibited *frustration* with what they perceived to be patients’ unreliability, lack of insight, and perceived resistance about their medications.
2. Clinicians felt *uncertain* about their competence to deprescribe.
3. System-level issues like care fragmentation and time pressure exacerbated feelings of overwhelm and confusion.

An additional subtheme that emerged within the oncologist group was *ambivalence* about deprescribing, due to perceived conflict with the physician’s role to treat with medications. Patients expressed a similar variation of this subtheme, believing that the physician’s role compelled them to prescribe the newest and “best” medications.

### Emotional facilitators of deprescribing

Subthemes related to emotional facilitators of deprescribing were also identified (Table 2 and Supplemental Table 2). All focus group participants expressed unprompted, spontaneous positive emotion toward pharmacists. Pharmacists were universally viewed as trustworthy, objective, and expert. They were also perceived as being “outside” of the role expectations and time pressures experienced by other clinicians. Pharmacists themselves expressed confidence in their abilities and role related to deprescribing. As a separate subtheme, pharmacists talked about their ability to negotiate the emotions invoked by deprescribing, and specifically noted their training and approach to communicating with physicians; these approaches included specific awareness of the emotional context of these discussions, and the need to mitigate adverse emotional reactions. Lastly, a facilitator subtheme revealed that unlike other clinicians, pharmacists more often situated their comments about patients within a nuanced understanding of the system complexity and patient experience within that system. These comments were typically expressed in an emotionally neutral way, in contrast to the negative emotional content around perceived patient-centered barriers expressed by oncologists, PCPs, and oncology nurses. Unsurprisingly, patients were also attuned to individual context and experience.

**Table 2:** Emotional facilitators of deprescribing

*Subtheme*	*Group*					*Example quotes*
	Onc	PCP	Nurse	Pharm	Pts	
Pharmacists as open, neutral experts	X	X	X	X	X	• Patient 1: “I think [pharmacists] would be one of the best people to do this, if in fact you gave them a list of everything you take both, um, over-the-counter and prescribed.” Patient 2: “I have done that sometimes because I realize that they don’t have a—um…” Patient 3: “Skin in the game.” Patient 2: “Yeah, they don’t have skin in the game. I mean, they can give you an independent opinion. So, even if—I always check it with the pharmacist, actually—even if the docs have said yeah.” (*Patients*) • “I bounce ideas off of them. Hey, is this okay. Does this interact. And they are always so open to answering questions.” (*Nurse*)
Emotionally aware communication				X		• “I think one of the things that we’re taught in pharmacy school is to take kind of that soft approach, because we never want to look like we’re overstepping. And so I was always taught when I make my recommendations, that it would be phrased as either a recommendation or ‘consider doing XYZ.’” (*Pharmacists*)
Understanding patient context				X	X	• “I think it just adds complexity to [patients] from my perspective of providing care to them, but also complexity to their life for having to manage all these different medications. It makes it more challenging for everyone that’s involved with their care, to make sure that they get all the ones that they need, that there’s no mistakes made, that they are on all the right things, that nothing interacts.” (*Pharmacist*) • "We have to be careful we’re not grouping everyone into the same category.” (*Patient*)

### Validation of results with emotion coding

Emotion coding revealed 161 text segments expressing negative emotion and 44 statements expressing positive emotion. The most common negative emotions expressed were frustration (*n*=33), being overwhelmed (*n*=23), and concern/alarm (*n*=16). The most common positive emotions expressed were appreciation (*n*=21), satisfaction (*n*=7), and pride (*n*=5). Most negative emotional statements were expressed by oncologists (*n*=57) and primary care physicians (*n*=49), followed by nurses (*n*=29), patients (n=15), and pharmacists (*n*=11). Primary care physicians had the highest number of positive emotional expressions (*n*=16) followed by oncologists (*n*=9), nurses (*n*=9), patients (*n*=6), and pharmacists (*n*=4).

## Discussion

In this manuscript, we explore emotional barriers and facilitators of deprescribing to address polypharmacy in older adults with cancer. Polypharmacy is very common in this population, and It is particularly challenging for those starting cancer treatment since chemotherapy regimens typically include the treatment agents themselves along with multiple supportive care medications (e.g., antiemetics). Cancer-related symptoms often require yet more medications. The oncology clinic provides many potential facilitators for addressing polypharmacy: a cancer diagnosis often prompts re-evaluation of goals of care and treatment priorities; oncologists see patients frequently, often assuming primary care roles for their patients, and pharmacists may be more accessible due to their role in evaluating and preparing cancer treatments. Deprescribing within the oncology clinic has been shown in a small pilot study to be feasible and well-accepted by patients [9]. However, data on implementation and outcomes in this context remain very sparse, outside of data surrounding end-of-life deprescribing. A cluster-randomized trial to evaluate and compare two implementation strategies for deprescribing has been developed and is currently accruing patients. Focus groups were conducted with oncologists, nurses, PCPs, pharmacists, and patients prior to trial initiation to provide feedback to refine implementation approaches. Participants unanimously recognized polypharmacy to be a significant issue. Although this may reflect self-selection bias in volunteering for the focus groups, participants expressed an awareness in medical literature and media surrounding polypharmacy in older adults.

Many of the barriers and facilitators emerging from these groups overlap with those previously described in the literature, including individual-level emotional factors such as fear and uncertainty [22, 36, 37]. The frequency of emotional responses in these focus groups, and particularly statements of negative emotion around deprescribing, is noteworthy: although negative emotions were expressed within the context of discussing real or perceived barriers to implementation (such as patient expectations or time pressure), the emotions themselves represent barriers to deprescribing.

Clinicians expressed multiple emotional barriers to deprescribing, including moral emotions about being judged by colleagues or patients, shame, frustration toward patients, and uncertainty. Both clinicians and patients expressed ambivalence about deprescribing, indicating that it conflicted with what they had been taught to expect from a healthcare interaction [38]. Text segments expressing a negative emotion were higher than positive expressions within these focus groups; although all groups stated that polypharmacy is a problem, all groups except pharmacists had difficulty envisioning interventions. Instead, there was a clear tendency to raise perceived barriers to intervention with signifiers of negative emotion including “I feel” statements, sarcasm, and shifts in intonation or volume. By contrast, pharmacists utilized neutral language and expression, endorsed self-confidence and expertise, acknowledged the need for emotionally aware communication around deprescribing, and recognized how patient context could affect their ability to understand medications.

Deimplementation in healthcare, of which deprescribing is an example, can provoke emotions and psychological biases in clinicians that hinder action [39, 40]. The psychological context and requirements of deimplementation differ substantially: deimplementation involves “unlearning,” which can induce emotional reactance that increases,rather than decreases, the commitment to the prior learning [41]. In the case of deprescribing, unease can result from re-evaluating and overturning prior decisions regarding the benefit of medications. Clinicians are susceptible to overvaluing their prior decisions (endowment effect), interpreting new evidence as reaffirming their previously held beliefs (confirmation bias), and prioritizing emotionally vivid feedback such as their experiences with a disgruntled patient in their decisions (availability heuristic) [14]. Deprescribing also challenges the therapeutic illusion favoring action over inaction in medicine [42]: it violates the role expectations of those who “heal with meds” [38] and the belief that “more” and “newer” are necessarily better. Finally, care fragmentation and time pressures resulting from healthcare delivery, organization, and financing, can amplify the effects of these biases. In these focus groups, this amplification could be detected in statements deferring or diffusing responsibility for deprescribing via expression of fears about “staying in one’s lane,” avoiding conflict with other prescribers, facing legal concerns, and anticipating patient reactions.

Patients are susceptible to similar psychological biases and expectations. However, a large survey study of Medicare beneficiaries revealed that two-thirds of older patients wanted to decrease their medication usage, and 92% were willing to stop medications if recommended by their physician [15]. Patients’ desire to de-implement other low-value interventions has also been shown in the context of the American Board of Internal Medicine (ABIM) Foundation’s Choosing Wisely campaigns [43], indicating that clinicians may be overestimating patient resistance to deimplementation. However, the time required to engage in education and “unlearning” with patients may be unrealistic in the setting of short clinic visits, particularly oncology visits where other discussions are prioritized.

Nonetheless, all participant groups arrived at a similar resolution for addressing not only systemic but psychological barriers to deprescribing: embracing pharmacists as the locus of deprescribing. Pharmacists were noted as trustworthy by all groups, and they expressed high self-efficacy in understanding and addressing nuanced perspectives around medication use in older adults. Transcript analysis revealed a shared understanding that pharmacists could mediate the cognitive dissonance exposed by the process of deprescribing, displacing this dissonance and its associated emotions onto an objective and expert third party. Helfrich et. al. suggest that a dual process model of cognition is applicable in this situation, whereby “unlearning” (reflective cognition) and substitution of an alternative action (automatic cognition) could work in concert to enhance de-implementation [41]. Pharmacists describe role behaviors supporting clinicians in both processes: they reflect and reframe information to clinicians, and they provide an alternative action for clinicians to perform (e.g., consulting the pharmacist). Furthermore, they help to offset the systemic factors such as time pressure and multitasking that inhibit clinicians’ reflective cognition and capacity for deimplementation [44]. In the focus group, pharmacists described awareness of the emotional impact of their roles, as well as specific training to allow them to navigate and mitigate this impact. Well-designed randomized trials focused on pharmacist-led interventions have successfully reduced high-risk medications in older adults without cancer [45, 46].

This study has several limitations. First, the focus groups were not specifically undertaken to elicit or understand emotions about deprescribing, and it is possible that thematic saturation was not reached. Second, different groups had somewhat different patterns of emotional expression (see Tables 1 and 2), and it is unclear whether these patterns are generalizable across similar groups (i.e., whether nurses consistently express different emotional reactions than oncologists, for example). We did not perform additional focus groups to evaluate these limitations. Third, most participants were recruited from a single institution, and across all groups the participants worked together and knew one another, which may have influenced their emotional expression. Finally, emotions are conveyed via tone of voice, facial expressions, body language, and other cues that cannot be detected in written transcripts. We augmented open coding analysis of the transcripts with contemporaneous notes, recorded videos, and member checking, but some emotional nuance still may have been lost as the focus groups were conducted via teleconferencing.

In conclusion, care providers working in an oncology setting and older adult patients with cancer express emotional reactions that signify potential barriers and faciltators to deprescribing. Emotional barriers include worry, fear, and ambivalence; facilitators include trust in pharmacist expertise, emotionally aware communication, and empathy to patient context. Despite a growing literature and associated guidelines supporting planned medication discontinuation, deprescribing remains an uncommon practice in the oncology clinic for older adults with cancer. Although this lack of uptake is related to multiple systemic and institutional barriers, the specific emotions induced by contemplation of deprescribing may be underappreciated barriers. How these emotions either inhibit or facilitate deprescribing behaviors is rarely explored in the literature. Deimplementation (such as deprescribing) invokes different emotional responses and biases compared to implementation (such as prescribing), which may create unique difficulties in designing interventions to reduce inappropriate medication use. Many of the emotional responses are linked to either common cognitive biases (such as overcommitment to one’s prior decision to initiate a medication) or to deeply acculturated values in medicine (such as healing with medications, or using the “newest and best” technologies). Utilizing solely logic- and data-based retraining is unlikely to address these emotional barriers. Instead, the focus groups as a whole suggest a different approach: the transference of some responsibility for deimplementation onto an “objective” and expert adjudicator: the clinical pharmacist. Due to their role and training, clinical pharmacists may not be subject to the same cognitive biases and role acculturation, and they may be able to assist clinicians in their own “unlearning” processes by providing reframing and substituted action. In our clinical trial, pharmacist-led deprescribing will be tested against a patient education brochure in the oncology clinic for older adults with polypharmacy initiating cancer treatment. The trial is designed with an adaptive “lead-in” period to refine and adapt the pharmacist intervention, to determine how best to utilize the pharmacist skillset to enhance facilitation of deprescribing and mitigate identified barriers. The cluster-randomized portion of the trial will collect both effectiveness and feasibility outcomes. Since clinical pharmacists are tightly integrated into oncology practice, this poses a scalable and effective mechanism for decreasing polypharmacy and improving outcomes in these patients. Further research is needed to address how deprescribing is optimally performed in older patients with cancer and polypharmacy [47].

### Supplementary information

ESM 1 (DOCX 21.4 kb)

ESM 2 (DOCX 21 kb)

ESM 3 (PDF 495 kb)

## Data Availability

The raw/processed data required to reproduce the above findings cannot be shared at this time due to anticipated difficulty de-identifying transcripts, and because the data also forms part of an ongoing study.
