# Supplementary material for: Emotional barriers and facilitators of deprescribing for older adults with cancer and polypharmacy: a qualitative study
Source: Support Care Cancer. 2023 Oct 17;31(11):636. doi: 10.1007/s00520-023-08084-9 (PMC10581937; doi:10.1007/s00520-023-08084-9)
Supplement: Supplementary file 1 — (DOCX 21.4 kb) [file 520_2023_8084_MOESM1_ESM.docx]

**Supplemental Table 1. Emotional barriers to deprescribing, additional quotes.**

| ***Subtheme*** | ***Group*** | | | | | ***Example quotes*** |
| --- | --- | --- | --- | --- | --- | --- |
|  | Onc | PCP | Nurse | Pharm | Pts |  |
| Apprehensive about colleague reactions | X | X |  | X |  | - “It’s hard to tell people to stop medications that other people are prescribing. I feel obligated to respect the other providers that were prescribing that medication.” (*Oncologist*) - “…the times that things go badly is usually when you miss something in the chart, which of course makes us look bad because and it can be a little bit embarrassing because it's like, ‘Oh they're not on that because of something that is obvious.’” (*Pharmacist)* |
| Preoccupied with patient reactions | X | X |  | X |  | - “My major concern would be that…the patient is going to look at this and say my doctors are idiots, look at all this stuff they have me on. I think that it could sow a lot of unnecessary doubt if there are good reasons to be on these things…it may inadvertently create a kind of antagonistic showdown down the road when they are trying to say, “Dr. So-and-so, why do you have me on an aspirin when there’s no role for this in somebody over the age of 80 and you have me on this, this and this. And I looked it up and are you trying to kill me?”’ (*Oncologist)* - “I had an interaction this week where someone was coming in for a physical and they’re like I want all that blood work repeated. I’m like you had it all done last year and it was perfectly normal. What? You want to save money for the insurance company? Are you worried about that? I’m like no, it’s a waste of time. You know. But they were very – you know, the abandonment was a real issue there.” (*PCP*) |
| Frustration toward patients | X | X | X |  |  | - “I still go through it with them but then they’re just – they’re just yes, yes. And I know a lot of times they are not even really listening to what I’m saying or what I’m asking them but they are so flustered that they take so many or they’ll just say, ‘I take a lot of pills. I don't know what they are for. The doctor told me to take them, I just take them. Yes, everything on there, yes.’ And they’re too flustered.” (*Nurse*) - PCP 1: “The ones that I want them to come off are the ones they want to stay on. The ones they want to get off are the ones they can’t get off.” PCP 2: “Vitamin C. [Laughter] Please.” (*PCP*) |
| Uncertain about competence | X | X | X |  |  | - “I wasn’t in the room when the oncologist was explaining everything. You know? They’re the one with the white coat. Not me. I’m a nurse in scrubs.” (*Nurse*) - “There are people that come in and I don't feel competent…first of all to understand why they are on some of these medications and whether I can stop them or not.” (*PCP*) - “I think a lot of times it’s just this nebulous “Huh, they are on five hypertensives and hmm, these seem to have a lot of overlay but these were prescribed by the cardiologist, this was by the nephrologist,” and so there’s a little hesitancy to figure out how one is authorized to simplify between all those different indications.” (*PCP)* - “It’s not really part of our education. We certainly learn about pharmacology and certainly know what we would prescribe for different disease processes and so forth but we don’t really have, looking at an overall basis of polypharmacy and what the interactions are.” (*PCP)* - “I’ll also add that I’m having a harder time, and I’m only a year out, of recognizing some of the names and knowing some of the drugs of these patients that they’re on, right?” (*Oncologist*) |
| Overwhelmed and confused by roles in a fragmented system | X | X | X |  |  | - “Doctors don’t have the time to continue with this. And nurses now either. Our responsibilities, you know, we have a lot. So somebody else to kind of help coach through these changes and their emotions…[w]hy they’re choosing to take their treatments. Why they don’t want to stop their medications. That’s all a big part of it, too.” (*Nurse*) - “And don’t forget the time element, too. I mean you’ve got 18 medicines. You’ve got your 20 minute visit if you’re lucky and you have many other priorities. Am I really going to go through that med list one by one? And they’re like is that the green pill?” (*PCP*) |
| Ambivalence about deprescribing based on expected role | X |  |  |  | X | - “It seems as if physicians look at their interaction with patients as successful if they wrote a prescription and feel like that’s the answer to the patient’s complaint.” (*Oncologist*) - “It is very seldom that you’ll have an interaction especially with a consultant, someone from a different field and all, who doesn’t want to write a prescription when you go away.” (*Oncologist)* |

**Supplemental Table 2. Emotional facilitators of deprescribing, additional quotes.**

| ***Subtheme*** | ***Group*** | | | | | ***Example quotes*** |
| --- | --- | --- | --- | --- | --- | --- |
|  | Onc | PCP | Nurse | Pharm | Pts |  |
| Pharmacists | X | X | X | X | X | - “Pharmacists should be the ones making the assessment of this and making recommendations for it.” (*Pharmacist*) - Oncologist 1: “If you get a pharmacist involved this is their area of expertise. I could see having a better buy in than from the folks that are actually prescribing these medications.” (*Oncologists*) - “I think using the pharmacist whether it be eConsult or if you are lucky enough to have access to one, I think it’s very important.’ (*PCP*) |
| Emotionally aware communication |  |  |  | X |  | - “So, that’s something that they train on in their third and their fourth year of pharmacy school, is getting used to presenting their ideas to providers. And then if they go on to do residency, as most of the people that have been on here have done, they again, another year or two of practice just getting comfortable accepting the fact that they are the medication expert.” (*Pharmacist)* - “Yeah, even if there truly is no indication for the medicine, I usually would write something like ‘no clear indication apparent based on review of problem lists,’ like kind of to allude to the fact that maybe I’m missing something. I think, as pharmacists, sometimes we run into the having to maybe be too nice, so I could be being overly cautious with this and we could just get over it and be right to the point, but I think it’s just something to consider.” (*Pharmacist*) - “I think across the system, almost all the clinical pharmacists that I’ve worked with have that ability to kind of tailor their approach based on the provider and maybe their style. I mean, I work with five different providers here in the office and how I might approach it it’s probably five different ways for each of them, just because as you get to know people and you see what they’re more likely to be receptive to.’ (*Pharmacist*) |
| Understanding patient context |  |  |  | X | X | - Pharmacist 1: “Yeah, I think the older adult it adds just like another layer of complexity with their age, given that then sometimes there’s like cognitive concerns, loss of dexterity, potentially, to use the different medications appropriately. So, I think, yeah, that just adds like a whole other layer to it. I think polypharmacy is complex in younger patients, but even worse, potentially, in older adults.” Pharmacist 2: “For me, one of the things that comes to mind is that we tend to deal with patients that are much more sensitive to side effects and drug interactions, as well.” Pharmacist 3: “I’d also say for our older adults some things get put on the list and don’t appropriately get taken off, which adds that complexity to it.” Pharmacist 4: “I think it’s really hard to understand what because it’s listed in the computer doesn’t mean the patient’s actually taking it that way. So, knowing what the patient’s actually doing at home versus just what they’re told is a big issue.” Pharmacist 5: “Yeah, and I also think that sometimes you get other people taking care of the older folks, so you may have them being responsible for the meds, while the patient themselves doesn’t necessarily know what they’re on, so that can add to the complexity as well.” Pharmacist 2: “They also have multiple providers, or are more likely than our younger population to have multiple providers involved. And…across multiple health systems, and translating that information safely can be very challenging and adds to that complexity.” (*Pharmacists*) - “Many patients don’t get all of their prescriptions at the same pharmacy, uh, especially now when you can order online, um, and when there is so much information about cost comparison. So, people sometimes get one medication at one pharmacy, a different medication somewhere else, uh, so it makes it, um, less reliable.” *(Patients)* |
