# Supplementary material for: Emotional barriers and facilitators of deprescribing for older adults with cancer and polypharmacy: a qualitative study
Source: Support Care Cancer. 2023 Oct 17;31(11):636. doi: 10.1007/s00520-023-08084-9 (PMC10581937; doi:10.1007/s00520-023-08084-9)
Supplement: Supplementary file 2 — (DOCX 21 kb) [file 520_2023_8084_MOESM2_ESM.docx]

# Supplemental Material

These questions exemplify the types of questions asked in the focus group and the themes to be explored; actual questions may deviate from the below during the focus groups.

# Group 1: Oncologists

1. I want to think about medication usage in older adults. What comes into your mind when I say that? What are some ways that medication usage differs in older adults compared to, say, younger people?
2. Tell me what you know about polypharmacy. <*If participants unfamiliar, give definition*> What is your experience with this in your patients? How do you identify when polypharmacy is occurring?
3. I want to hear about how you do medication management in your practice. *Probes: Who asks about medications? Is it a formal part of clinic workflow? Do you discuss medication use as part of the clinic visit?*
4. If someone were coming up with a standard way to help older adults with polypharmacy, what might that look like? *Probes: Who would need to be involved? Where could an intervention be done? What resources are needed? What would your involvement be?*
5. Now I want to switch topics and think more generally about how you like clinical data communicated to you. For example, you send someone for a specialist evaluation – what most helps you to understand their evaluation and recommendations?
6. Let’s look at a sample written template for sharing recommendations about medication management in older adults. <*Show example communication template for EMR communication >* I am going to give you a couple of minutes to read this and please jot some notes. I’m interested to hear what you think about this. *Probes: What do you like? What do you dislike? What would you change?*
7. We are going to wrap up now. Thank you again for your time and energy. My last question is, when we write up our report for this group, what is one important point that we should pay attention to when it comes to addressing polypharmacy in older adult?

# Group 2: Clinical Pharmacists

1. Let’s think about medication usage in older adults. What comes into your mind when I say that? *Probes: does medication usage differ in older versus younger people? How? Can you tell me about an experience that struck you about medication usage in an older person?*
2. What is ‘polypharmacy’, in your experience? *Probes: How do you know when it is occurring? How many medications are too many? Do you use tools to assess this, or is it more of a gestalt?*
   1. If we were screening older adults for polypharmacy in a standard way, what would be the best way to do that?
   2. I want to share an example of a possible screening tool for polypharmacy. I will give you a moment to really look at this *<show screening tool>*. What are your thoughts about it?
3. Now that we have thought a bit about how we might identify older adults with polypharmacy, I want to shift and think about what we might do about it once we identify it. What do you think should be done to address it? *Probes: Should we be intervening for polypharmacy? Who should be involved? What should the pharmacist’s role be?*
4. Now, we are going to shift to a different topic. I want you to think about a time when you had to communicate recommendations or concerns to a clinician. When did that go best? When did it not go so well? How do you prefer to communicate with clinicians? Can you describe a situation where you noticed a potentially unsafe medication on a patient’s list? Specifically, what did you do about it? How was the information received?
5. I now want to show you one way to communicate information and recommendations about medication to a clinican. *<show template>.* I am going to give you a couple of minutes to read this and please jot some notes. I’m interested to hear what you think about this. *Probes: What do you like? What do you dislike? What would you change?*
6. We are going to wrap up now. Thank you again for your time and energy. My last question is, when we write up our report for this group, what is one important point that we should pay attention to when it comes to addressing polypharmacy in older adults?

**Group 3: Oncology nurses**

1. We are going to start by talking about medication usage in older adults. What comes into your mind when I say that? Probes: does medication usage differ in older versus younger people? How? Can you tell me about an experience that struck you about medication usage in an older person?
2. Can you tell me about your role when it comes to medications in older patients? Probes: Are you involved in asking about medications for patients? What is the workflow like? Does everyone do this the same way?
3. Now we are going to shift to a new topic. Think about a time when you had a concern about one of your patients, and you weren’t sure the oncologist knew about it. This experience could be related to medications, or any other concern. How did you communicate this concern? How was it received? Did you talk directly to the patient about your concern?
   1. Now think specifically about if you had a concern related to medications. What would you do about that concern?
   2. Can you talk a bit about your level of comfort addressing a medication concern on your own with the patient?
4. I now want to show you one way to communicate information and recommendations about medication might be communicated to the oncology team. <show template above. I am going to give you a couple of minutes to read this and please jot some notes. I’m interested to hear what you think about this. Probes: What do you like? What do you dislike? What would you change?
5. I know many of you also give recommendation and education to patients. I want you to think about a time you were educating a patient, particularly an older adult, about their medications. What was important in this process? What didn’t work well?
6. We are going to wrap up now. Thank you again for your time and energy. My last question is, when we write up our report for this group, what is one important point that we should pay attention to when it comes to addressing polypharmacy in older adults?

**Group 4: Primary Care Physicians (PCP’s)**

1. I want to think about medication usage in older adults. What comes into your mind when I say that? What are some ways that medication usage differs in older adults compared to, say, younger people?
2. Tell me what you know about polypharmacy. <*If participants unfamiliar, give definition*> What is your experience with this in your patients? How do you identify when polypharmacy is occurring?
3. I want to hear about how you do medication management in your practice. *Probes: Who asks about medications? Is it a formal part of clinic workflow? Do you discuss medication use as part of the clinic visit?*
4. If someone were coming up with a standard way to help older adults with polypharmacy, what might that look like? *Probes: Who would need to be involved? Where could an intervention be done? What resources are needed?What would your involvement be?*
5. Now I want to switch topics and think more generally about how you like clinical data communicated to you. For example, you send someone for a specialist evaluation – what most helps you to understand their evaluation and recommendations?
   1. What are your thoughts and feelings about specialists taking “direct action” with your patients? What do you want to happen when a specialist has recommendations – directly address it, discuss with you first, let you undertake the recommendations?
   2. Let’s say someone recommended your patient stop a medication. Tell me your thoughts about that. *Probes: would you want the patient to discuss with you first? Are you comfortable with other physicians discontinuing medications for your patients? In what circumstances?*
6. Let’s look at a sample written template for sharing recommendations about medication management in older adults. <*Show template>* I am going to give you a couple of minutes to read this and please jot some notes. I’m interested to hear what you think about this. *Probes: What do you like? What do you dislike? What would you change?*
7. We are going to wrap up now. Thank you again for your time and energy. My last question is, when we write up our report for this group, what is one important point that we should pay attention to when it comes to addressing polypharmacy in older adults?

**Group 5: Patient Advocates**

1. Today, the topic we are talking about is older adults who take too many medications. To get started, I want each of you to think about someone you know who is taking too many medications. If you don’t know anyone like this, think about what you have heard from friends or family about medication concerns. Take a few minutes and jot down some things you have heard, or notes for a story about taking too many medications. We will go around the group and share these reflections.
2. Let’s examine these stories and reflections and pick out the things in those stories that are part of this idea of “taking too many medications.” What things are connected to this idea? What are we missing that is connected to this idea?
3. Now I want you to step back and think about if you were taking too many medications, whatever that means to you. Perhaps you have already personally experienced this, or are experiencing it now. What would be important to know about you and your experience? *Probes: How would you like someone to address your concerns about this? Who should that person be? What is important when it comes to deciding how many medications is “too many?”*
4. I have sent you 2 brochures in advance of this focus group. I want you to pull them out now and spend a few minutes examining them. Jot down some notes about what you like, what you don’t like. I then want to discuss which you prefer, and why. How should we change them?
5. We are going to wrap up now. Thank you again for your time and energy. My last question is, when we write up our report for this group, what is one important point that we should pay attention to when it comes to thinking about older adults taking too many medications?
